# Social inequalities in self-perceived health in Chile, does the urban environment matter?: a cross-sectional study

**DOI:** 10.1186/s13690-023-01136-w

**Published:** 2023-07-07

**Authors:** Natalia López-Contreras, Vanessa Puig-Barrachina, Alejandra Vives, Paola Olave-Müller, Mercè Gotsens

**Affiliations:** 1 Universitat Pompeu Fabra, Barcelona, Spain; 2 Agència de Salut Pública de Barcelona, Barcelona, Spain; 3 Universidad de La Frontera, Temuco, Chile; 4 Institut d’Investigació Biomèdica Sant Pau (IIB SANT PAU), Barcelona, Spain; 5 Departamento de Salud Pública, Escuela de Medicina, & Centro de Desarrollo Urbano Sustentable, CEDEUS, Pontificia Universidad Católica de Chile, Santiago, Chile

**Keywords:** Urban health, Social Determinants of Health, Health Status indicators

## Abstract

**Background:**

The health of a population is determined by urban factors such as the physical, social and safety environment, which can be modified by urban regeneration policies. The aim of this study was to analyze the associations of elements of the social, physical and safety environment of the neighborhood in the urban context with self-perceived health (SPH), according to axes of inequality, such as gender and educational level in Chile in 2016.

**Methods:**

Cross-sectional study using a nationally representative population-based survey of Chile. We used data from the 2016 National Survey of Quality of Life and Health. Poor SPH in the urban population older than 25 years was analyzed in relation to social, physical and safety environment variables. Poisson multilevel regression models were estimated to obtain prevalence ratios (PR) and their respective 95% confidence intervals (95%CI). All analyses were stratified by sex and educational level.

**Results:**

SPH was worse in women than in men, especially in those with a lower education level. Poor SPH was associated with lack of support networks (PR = 1.4; 95%CI = 1.1–1.7), non-participation in social organizations (PR = 1.3; 95%CI = 1.1–1.6) and perceived problems with the quality of public space (PR = 1.3; 95%CI = 1.2–1.5) in women with a medium-high educational level and with a feeling of not belonging to the neighborhood (PR = 1.5; 95%CI = 1.2–1.8) and the perception of pollution problems (PR = 1.2; 95%CI = 1.0-1.4) in women with a low educational level. A feeling of unsafety was associated with both educational levels (PR = 1.3; 95%CI = 1.0-1.5). Poor SPH was associated with the feeling of not belonging (PR = 1.7; 95%CI = 1.2–2.5), and unsafety (PR = 2.1; 95%CI = 1.8–2.4) in men with a medium-high educational level, while there were fewer associations in men with a lower education level.

**Conclusions:**

Urban interventions are recommended to improve the health of the resident population and should take into account axes of inequality.

**Table:** 

**Text box 1. Contributions to the literature**
• Research has demonstrated the importance of conducting analyses on social determinants based on relevant axes of inequality such as gender and socioeconomic status, as they reveal differences that can be explained by individual and contextual factors in their living environments.
• These findings address the existing gaps in the literature, including stratified analyses by gender and socioeconomic position, and provide direct input for policies aiming to integrate health and urban planning.
• The findings contribute to expanding the knowledge in the context of countries in the global South and encourage reflection in relation to the limited studies conducted on these countries.

## Introduction

People’s health and quality of life are influenced by multiple social determinants [1] – the circumstances in which people are born, grow, live, work, and age–, some of which are related to neighborhood conditions [2]. In cities, urban social determinants are related to elements such as the physical and socioeconomic environment, including urban planning, housing, transport mobility and the quality of air, as well as employment and working conditions, social networks and community participation. These elements are, at the same time, shaped by the urban governance, that is, the distribution of political power of the local and national government and other key actors in the private sector as well as civil society [3].

According to the conceptual framework developed by Borrell et al. [3]., which examines health inequalities in European cities, there is an unequal distribution of the quality of physical and socioeconomic elements, producing health inequalities – i.e. avoidable, unfair and systematic differences in health between different groups of people [4] –. Thus, health inequalities tend to be more marked in urban areas because of the effect of segregation, by which in some neighborhoods factors of deprivation and poor populations are concentrated. Although this conceptual framework examines health inequalities in European cities, it provides insights that may be applicable to urban contexts in other regions.

Regarding, factors related to the social environment, such social interaction in neighborhoods and participation in community activities, are associated with better health. In this sense, European and Anglo-Saxon studies have shown that good self-perceived health (SPH) is associated with higher levels of social participation [5], a feeling of belonging to the neighborhood [6], and access to support networks [7]. Likewise, factors related to the physical environment of the neighborhood, such as access to neighborhood infrastructure and green areas, have been associated with healthy behaviors such as physical activity [8] and better mental health outcomes [9]. On the other hand, the perception of unsafety, which can be related to both poorly lit spaces and criminal behavior in the neighborhood, also affects health and quality of life [10,11], by limiting movement outside the home, which can decrease social interaction and physical activity [11].

The effect of neighborhood environments also depends on some axes of inequality such as gender and socioeconomic position. In relation to gender, positive associations have been described between SPH and elements of the social and physical environment of the neighborhood in women, such as high trust in neighbors, and better physical quality of the residential environment [12]. In addition, compared with men, women have a greater perception of unsafety and, consequently, experience fear in poorly-lit neighborhoods or those with corners where people can hide, associated with street sexual harassment [13], which has a negative impact on their health [14]. In relation to socioeconomic status, the literature indicates that people with lower socioeconomic status show less social participation [15], and have more safety concerns about the neighborhood, which are negatively associated with physical activity, mental health and SPH [16].

Chile is a country located in Latin America, with a large urban population (87.7% according to the 2017 Census 17), and with deep socioeconomic inequalities between its different administrative divisions, such as regions or cities [18], which are manifested in health inequalities. Thus, studies have reported higher infant [19] and total mortality [20,21] in poorer regions and, at the individual level, higher mortality among people with fewer years of schooling [22]. In this context of inequalities, and based on the scientific knowledge that improvements in the neighborhood environment could enhance inhabitants’ quality of life, urban planning policies have been designed and implemented in Chile during last 15 years. These programs affect various elements of the neighborhood, which could have repercussions on the health of the population, which could, in turn, differ by gender or socioeconomic position. However, there is no evidence on the relationship between the main factors of the neighborhood environment and health in Chile, or whether these relationships are modified by gender and socioeconomic position. Therefore, the aim of this study was to analyze the associations of elements of the social, physical and safety environment of the neighborhood in the urban context with SPH, according to axes of inequality, such as gender and educational level in Chile in 2016.

## Methods

### Design and information source

A cross-sectional study was conducted using data from the latest 2016 Quality of Life and Health Survey (in Spanish: “Encuesta de Calidad de Vida y Salud” [ENCAVI]). ENCAVI is a face-to-face household survey of people aged 15 years and older who usually reside in the urban and rural areas of the 15 regions of Chile (n = 7041). The sample design was probabilistic, geographically stratified and multistage (four stages: communes, blocks, housing and individual). The participation rate was 78.7%^23^. For this study, respondents younger than 25 years were not included in the analysis, since their education was still in progress, nor was the rural population, since this is an analysis of urban social determinants. The sample of people over 25 years of age residing in an urban context consisted of 4992 people. A total of 14.1% of participants had missing data in the study variables, which were randomly distributed according to sociodemographic variables, and were therefore excluded from the sample. Consequently, the final sample consisted of 4257 people.

### Variables

SPH was used as a dependent variable because it is a good indicator of health status (in relation to quality of life, diagnosed diseases and physical functioning), health service utilization and mortality [23–25].

For the independent variables of the social, physical and safety environment of the neighborhood, the ENCAVI questions were used (Table 1), which are related to the conceptual framework of “Determinants of health inequalities in European cities” [3]. Some elements of the social environment related *to neighborhood belonging, support networks*, and *participation* were evaluated, divided into three categories, [26] depending on whether the element benefits the same person (*egotropic*), a group (*sociotropic*), or is *religious*. In addition, some elements of the neighborhood’s physical environment were evaluated, such as the *quality of spaces, pollution and the availability of neighborhood infrastructure*. Finally, safety was evaluated through a question related to *perceived neighborhood safety*.

**Table 1:** Description of Quality of Life and Health Survey (in Spanish: “Encuesta de Calidad de Vida y Salud”) variables used

Variable	Original question	Recategorization
*Dependent Variable*		
**Self-perceived health**	“Would you say that your health is: Excellent. Very Good. Good. Fair. or Poor?“	*“Good health”*: excellent. very good. or good. “*Poor health”*: fair or poor
*Social Environment Independent Variables*		
**Sense of belonging to the neighborhood**	“How much do you agree with the following statements: b) I feel that I belong to this neighborhood: Strongly Agree. Agree. Neither Agree nor Disagree. Disagree. or Strongly Disagree.“	*“Agree”*: Strongly Agree. Agree. *“Neither Agree nor Disagree”*: Neither Agree nor Disagree. *“Disagree”*: Disagree. or Strongly Disagree.
**Perception of support networks**	“How much do you agree with the following statements: i) I think my neighbors could help me in an emergency: Strongly Agree. Agree. Neither Agree nor Disagree. Disagree. or Strongly Disagree”.	*“Agree”*: Strongly Agree. Agree. Agree. or Strongly Disagree. *“Neither Agree nor Disagree”*: Neither Agree nor Disagree. *“Disagree”*: Disagree or Strongly Disagree
**Participation**	“Do you actively or frequently participate in the following organizations (participate in meetings and other activities at least once a month)” **Egotropic participation**: Sports or recreational club; artistic. cultural. women’s. senior citizen or support group identity. **Sociotropic participation**: Neighborhood groups; territorial associations or volunteer groups. **Religious participation**: Church	*“Yes participates”* if the person participates in at least one organization in the corresponding category. *“Does not participate”* if the person does not participate in any organization in the category.
*Physical Environment Independent Variables*		
**Perception of problems with quality of public space**	“What infrastructure and equipment problems do you identify in your neighborhood or locality? f) Insufficient or poorly maintained paving; i) Lack of or poorly cleaned streets and sidewalks; e) Poor lighting	*“No problem”* if the person does not report any of the problems. *“Yes Problem”* if the person reports at least one of the listed problems.
**Perception of pollution problems in the environment**	“What problems related to pollution or deterioration of the environment do you identify in your neighborhood or locality? (a) Noise nuisance; (e) Bad smells; (f) Micro garbage dumps; (h) Stray dogs”	*“No Problem”* if the person does not report any of the problems. *“Yes Problem”* if the person reports at least one of the listed problems.
**Perception of problem of infrastructure availability in the neighborhood**	“What infrastructure and equipment problems do you identify in your neighborhood or locality? a) Lack of squares. green areas. others; b) Lack of sports infrastructure (courts. gymnasiums. tracks. circuits. others); c) Lack of community centers. places for social gathering or recreation”	*“No problem”* if the person reports none of the problems *“Yes Problem”* if the person reports one of the problems listed.
*Independent Safety Variable*		
**Perceived safety in the neighborhood**	“How safe do you feel walking alone in your neighborhood when it is dark? Very unsafe. somewhat unsafe. somewhat safe. very safe”	*“Safe”*: Very safe *“Somewhat safe”*: Somewhat safe *“Unsafe”*: Very unsafe. Somewhat unsafe

Sex and educational level were used as stratification variables. Sex was used in two categories “male” and “female”, as a proxy for gender. Educational level was employed to measure socioeconomic position, recategorized as “low educational level”, in individuals with 8 or less years of schooling, and “medium/high educational level”, in those with 9 or more years of schooling. This categorization was chosen because, in the Chilean context, the 8-year cutoff has been seen as a good indicator of socioeconomic position in relation to mortality [27]. Age was used as a continuous variable and as an adjustment variable.

### Data analysis

First, a univariate analysis was conducted of the dependent variable and the independent variables. Second, a bivariate analysis was performed between poor SPH and each of the independent variables and was assessed for differences using the Chi square test. Third, to analyze the association between poor SPH and the conditions of the social and physical environment and safety, four multilevel Poisson models with robust variance were constructed to control for the hierarchical structure of the data, considering the respondents’ region of residence. The models were adjusted for age and allowed estimation of prevalence ratios (PR) with their respective 95% confidence intervals (95%CI). Model 1 determined the association between poor SPH and each independent variable separately. Model 2 examined the association between poor SPH and variables related to the social environment. Model 3 identified the association between poor SPH and physical environment variables. Model 4 assessed the association between poor SPH and all independent variables related to the social, physical, and safety environment.

All analyses were performed in STATA ® 15.1 considering the complex sample design including sample weight [28] and were stratified by sex and educational level.

## Results

The study variables are described in Table 2. The proportion of men (49.1%) and women (51.0%) was similar. Analysis of educational level showed that there was a higher proportion of men and women with middle/high schooling (men = 81.5%, women = 74.4%). The mean age was 47 years and was higher among men and women with a lower educational level. The prevalence of poor SPH was 19.9% in men and 35.1% in women. Analysis of the social environment variables showed that, in both men and women, most reported *feeling belonging to the neighborhood* and *perceiving support networks* and not *participating in any egotropic, sociotropic or religious organizations*. Analysis of the physical environment variables showed that most respondents reported problems in the *perception of problems with the quality of public space*, and in the *availability of infrastructure and equipment in the neighborhood*. In most of the environments, reports of worse conditions, both in the social and physical environment, were concentrated in people with fewer years of education. Perception of safety differed by sex; the prevalence of *unsafety* was 24.4% in men and 31.5% in women.

**Table 2:** Descriptive analysis of self-perceived poor health and variables related to social. physical and security environment according to sex and educational level. Chile 2016

			Men	Men with low educational level	Men with medium/high educational level	Women	Women with low educational level	Women with medium/high educational level
			n (%)	n (%)	n (%)	n (%)	n (%)	n (%)
	Total		2109 (49.1)	391 (18.6)	1718 (81.5)	2190 (51.0)	560 (25.6)	1630 (74.4)
	Age*		47 (65.8)	56 (123.5)	45 (70.8)	48 (53.0)	57 (102.2)	45 (61.3)
	Self-perceived health	*Good health*	1689 (80.1)	252 (64.5)	1437 (83.6)	1422 (64.9)	246 (43.9)	1176 (72.1)
		*Poor health*	420 (19.9)	139 (35.5)	281 (16.4)	769 (35.1)	314 (56.1)	454 (27.9)
*Social Environment*	Sense of Belonging to the neighborhood	*Agree*	1454 (69.0)	253 (64.6)	1202 (70.0)	1515 (69.2)	404 (72.0)	1112 (68.2)
		*Neither Agree nor Disagree*	465 (22.0)	95 (24.4)	370 (21.5)	413 (18.9)	83 (14.8)	330 (20.3)
		*Disagree*	190 (9.0)	43 (11.0)	147 (8.5)	262 (12.0)	74 (13.2)	188 (11.6)
	Perception of Support Networks	*Agree*	1462 (69.4)	244 (62.2)	1219 (71.0)	1615 (73.7)	407 (72.6)	1208 (74.1)
		*Neither Agree nor Disagree*	385 (18.3)	69 (17.6)	316 (18.4)	349 (15.9)	77 (13.8)	272 (16.7)
		*Disagree*	261 (12.4)	79 (20.2)	182 (10.6)	226 (10.3)	76 (13.5)	150 (9.2)
	Egotropic Participation	*Yes participates*	660 (31.3)	107 (27.4)	553 (32.2)	331 (15.1)	87 (15.5)	244 (15.0)
		*Does not participate*	1449 (68.7)	284 (72.6)	1164 (67.8)	1860 (84.9)	474 (84.5)	1386 (85.1)
	Sociotropic Participation	*Yes participates*	508 (24.1)	78 (19.8)	431 (25.1)	624 (28.5)	143 (25.4)	482 (29.5)
		*Does not participate*	1600 (75.9)	314 (80.2)	1287 (74.9)	1566 (71.5)	418 (74.6)	1149 (70.5)
	Religious Participation	*Yes participates*	254 (12.0)	67 (17.0)	187 (10.9)	442 (20.2)	137 (24.4)	305 (18.7)
		*Does not participate*	1855 (88.0)	325 (83.0)	1530 (89.1)	1748 (79.8)	423 (75.6)	1325 (81.3)
*Physical Environment*	Perception of problems with Quality of public space	*No problem*	1031 (48.9)	170 (43.4)	861 (50.2)	1024 (46.8)	233 (41.6)	791 (48.5)
		*Yes Problem*	1078 (51.1)	222 (56.6)	856 (49.9)	1166 (53.2)	327 (58.4)	839 (51.5)
	Perception of pollution problems in the environment	*No problem*	744 (35.3)	94 (24.1)	650 (37.8)	691 (31.5)	159 (28.4)	532 (32.6)
		*Yes Problem*	1365 (64.7)	297 (75.9)	1068 (62.2)	1500 (68.5)	401 (71.6)	1099 (67.4)
	Perception of problem of infrastructure availability in the neighborhood	*No problem*	798 (37.9)	123 (31.4)	675 (39.3)	794 (36.2)	179 (32.0)	615 (37.7)
		*Yes Problem*	1311 (62.2)	268 (68.6)	1042 (60.7)	1397 (63.8)	381 (68.0)	1015 (62.3)
*Safety*	Perceived safety in the neighborhood	*Safe*	684 (32.5)	100 (25.4)	585 (34.0)	588 (26.8)	152 (27.1)	436 (26.7)
		*Somewhat safe*	911 (43.2)	154 (39.5)	756 (44.0)	912 (41.7)	218 (39.0)	694 (42.6)
		*Unsafe*	514 (24.4)	137 (35.1)	376 (21.9)	691 (31.5)	190 (34.0)	500 (30.7)

* Mean and standard deviation

Differences in the prevalence of poor SPH according to the distinct independent variables are shown in Table 3. Among men overall, the prevalence of poor SPH was 36.0% among those reporting a *sense of not belonging*, 22.6% in those reporting *problems with the availability of neighborhood infrastructure* and 27.9% in those reporting *perceived unsafety*. These relationships were replicated in men with higher educational level. For men with a lower educational level, no differences in poor SPH were seen in any of the variables. Among women overall, statistically significant differences were observed in the distribution of poor SPH in most of the variables in the environments analyzed. Of note was the finding that the prevalence of poor SPH was 50.5% among women not perceiving *support networks*, 36.8% among those reporting *no religious participation*, 40.7% in those perceiving *problems in the quality of public space* and 38.7% in those reporting *problems of pollution in the environment*. These associations were maintained in women with medium/high educational level. Among women with less schooling, 74.5% of those reporting a feeling of *not belonging* had poor SPH. In all women, a gradient in poor SPH was observed with the *perception of unsafety*, with the prevalence of poor SPH being 66.7% among women with less education who perceived insecurity in the neighborhood.

**Table 3:** Prevalence of self-perceived poor health according to social. physical and safety environment variables. stratified by sex and educational level. Chile 2016

			Men		Men with low educational level		Men with medium/high educational level		Women		Women with low educational level		Women with medium/high educational level	
			**%**		**%**		**%**		**%**		**%**		**%**	
*Social Environment*	Sense of Belonging to the neighborhood	*Agree*	**19.0**	*	31.8		**16.3**	*	**33.7**	*	**53.7**	*	26.4	
		*Neither Agree nor Disagree*	**16.2**		34.5		**11.5**		**34.1**		**51.6**		29.7	
		*Disagree*	**36.0**		59.6		**29.0**		**45.0**		**74.5**		33.4	
	Perception of Support Networks	*Agree*	18.5		30.4		16.1		**33.4**	**	56.8		**25.5**	*
		*Neither Agree nor Disagree*	20.4		42.8		15.5		**32.9**		42.5		**30.2**	
		*Disagree*	27.4		45.0		19.8		**50.5**		66.2		**42.6**	
	Egotropic Participation	*Yes participates*	16.4		28.7		14.0		31.7		61.5		21.1	
		*Does not participate*	21.5		38.1		17.5		35.7		55.1		29.1	
	Sociotropic Participation	*Yes participates*	18.3		32.0		15.8		32.9		55.2		26.3	
		*Does not participate*	20.4		36.4		16.6		36.0		56.4		28.5	
	Religious Participation	*Yes participates*	20.1		35.1		14.8		**28.4**	*	51.4		**18.1**	*
		*Does not participate*	19.9		35.6		16.5		**36.8**		57.7		**30.1**	
*Physical Environment*	Perception of problems with Quality of public space	*No problem*	18.4		33.7		15.4		**28.7**	**	52.2		**21.8**	**
		*Yes Problem*	21.3		37.0		17.3		**40.7**		58.9		**33.6**	
	Perception of pollution problems in the environment	*No problem*	**15.9**	*	28.6		14.1		**27.3**	**	**46.1**	*	**21.6**	*
		*Yes Problem*	**22.1**		37.7		17.8		**38.7**		**60.1**		**30.9**	
	Perception of problem of infrastructure availability in the neighborhood	*No problem*	**15.5**	*	33.3		**12.3**	*	**31.2**	*	52.8		24.9	
		*Yes Problem*	**22.6**		36.6		**19.0**		**37.3**		57.7		29.7	
*Safety*	Perceived safety in the neighborhood	*Safe*	**12.7**	**	25.9		**10.5**	*	**25.4**	**	**41.8**	**	**19.7**	**
		*Somewhat safe*	**20.8**		39.0		**17.1**		**34.2**		**56.8**		**27.1**	
		*Unsafe*	**27.9**		38.7		**23.9**		**44.5**		**66.7**		**36.1**	

* p < 0.005

** p < 0.001

Tables 4 and 5 show the prevalence ratios of poor SPH in relation to the variables of social environment, physical environment and safety, adjusted by age. In men (Table 4) in model 1, the factors associated with the probability of poor SPH were *feeling of not belonging* (PR = 2.3; CI = 1.5–3.4) and *not perceiving support networks* (PR = 1. 6; CI = 1.1–2.3), *perception of pollution problems* (PR = 1.5; CI = 1.3–1.7), *perception of infrastructure availability problems* (PR = 1.5; CI = 1.3–1.7) and *perception of unsafety* (PR = 2.2; CI = 1.8–2.6). These trends were observed at both educational levels of the study. The associations were maintained on introduction of all the social environment variables in Model 2. In Model 3 (all physical environment variables), there was a significant association with the *perception of pollution problems*. The associations were generally maintained on introduction of all the variables (model 4), although not all were statistically significant. When stratified by educational level, men with middle/higher education who had a *feeling of not belonging* (RR = 1.7; CI = 1.2–2.*5), perceived problems of infrastructure availability* (RR = 1.6; CI = 0.9–2.9) and *perceived insecurity* (RR = 2.1; CI = 1.8–2.4) had a higher probability of poor SPH. Meanwhile, in men with less education, poor SPH was associated with the *not perceiving support networks* (PR = 1.4; CI = 0.9–2.2) and *feeling of not belonging* (RR = 1.7; CI = 0.9–3.4).

**Table 4:** Association between self-perceived poor health and social. physical and safety environment variables. men according to educational level. Chile 2016

				Model 1			Model 2			Model 3			Model 4	
			**Men**	**Men with low educational level**	**Men with medium/high educational level**	**Men**	**Men with low educational level**	**Men with medium/high educational level**	**Men**	**Men with low educational level**	**Men with medium/high educational level**	**Men**	**Men with low educational level**	**Men with medium/high educational level**
			PR (95%CI)	PR (95%CI)	PR (95%CI)	PR (95%CI)	PR (95%CI)	PR (95%CI)	PR (95%CI)	PR (95%CI)	PR (95%CI)	PR (95%CI)	PR (95%CI)	PR (95%CI)
*Social Environment*	Sense of Belonging to the neighborhood	Agree	1	1	1	1	1	1				1	1	1
		Neither Agree nor Disagree	1.1 (0.8–1.5)	1.3 (0.8–2.2)	0.9 (0.7–1.3)	1.0 (0.7–1.3)	1 (0.6–1.7)	0.9 (0.6–1.2)				0.9 (0.6–1.2)	1.0 (0.6–1.7)	0.8 (0.6–1.2)
		Disagree	2.3 (1.5–3.4)	2.1 (1.1-4.0)	2.3 (1.5–3.6)	2.0 (1.4–2.8)	1.8 (0.9–3.5)	2.2 (1.5–3.1)				1.7 (1.2–2.4)	1.7 (0.9–3.4)	1.7 (1.2–2.5)
	Perception of Support Networks	Agree	1	1	1	1	1	1				1	1	1
		Neither Agree nor Disagree	1.3 (1.1–1.6)	1.6 (0.9–2.9)	1.2 (1.0-1.4)	1.3 (1.2–1.5)	1.7 (1.0-2.7)	1.1 (0.9–1.3)				1.3 (1.1–1.5)	1.6 (0.9–2.9)	1.1 (0.8–1.4)
		Disagree	1.6 (1.1–2.3)	1.7 (1.2–2.4)	1.4 (0.8–2.5)	1.3 (1.0-1.8)	1.4 (0.9–2.1)	1.2 (0.8–1.9)				1.2 (0.9–1.7)	1.4 (0.9–2.2)	1.1 (0.6–1.8)
	Egotropic Participation	Yes participates	1	1	1	1	1	1				1	1	1
		Does not participate	1.1 (0.8–1.5)	1.2 (1.0-1.4)	1.1 (0.8–1.6)	1.0 (0.8–1.4)	1.0 (0.8–1.3)	1.0 (0.7–1.5)				1.0 (0.8–1.4)	1.0 (0.8–1.2)	1.0 (0.7–1.5)
	Sociotropic Participation	Yes participates	1	1	1	1	1	1				1	1	1
		Does not participate	1.2 (0.9–1.6)	1.2 (0.6–2.2)	1.2 (0.9–1.5)	1.1 (0.9–1.4)	1.2 (0.6–2.1)	1.1 (0.8–1.4)				1.1 (0.8–1.3)	1.1 (0.6–2.1)	1.0 (0.8–1.3)
	Religious Participation	Yes participates	1	1	1	1	1	1				1	1	1
		Does not participate	1.0 (0.7–1.6)	0.9 (0.4–1.9)	1.2 (0.7–2.1)	1.0 (0.7–1.5)	1.0 (0.6–1.9)	1.2 (0.7–1.9)				1.1 (0.8–1.7)	1.1 (0.6-2.0)	1.3 (0.8–2.2)
*Physical Environment*	Perception of problems with Quality of public space	No problem	1	1	1				1	1	1	1	1	1
		Yes Problem	1.3 (1.0-1.6)	1.2 (0.9–1.5)	1.2 (0.9–1.7)				1.0 (0.7–1.4)	1.1 (0.8–1.5)	1.0 (0.5–1.7)	0.9 (0.7–1.3)	1.1 (0.8–1.5)	0.9 (0.5–1.5)
	Perception of pollution problems in the environment	No problem	1	1	1				1	1	1	1	1	1
		Yes Problem	1.5 (1.3–1.7)	1.4 (0.8–2.2)	1.4 (1.2–1.7)				1.3 (1.1–1.6)	1.3 (0.8–2.2)	1.2 (1.0-1.5)	1.2 (1.0-1.5)	1.2 (0.7–2.2)	1.1 (0.8–1.4)
	Perception of problem of infrastructure availability in the neighborhood	No problem	1	1	1				1	1	1	1	1	1
		Yes Problem	1.5 (1.2–1.9)	1.1 (0.9–1.4)	1.7 (1.2–2.4)				1.4 (1.0–2.0)	1.0 (0.7–1.4)	1.6 (0.9-3.0)	1.4 (1.0-1.8)	0.9 (0.6–1.4)	1.6 (0.9–2.9)
*Safety*	Perceived safety in the neighborhood	Safe	1	1	1							1	1	1
		Somewhat safe	1.6 (1.2–2.1)	1.4 (0.9–2.3)	1.6 (1.1–2.3)							1.5 (1.1–1.9)	1.2 (0.7–2.2)	1.6 (1.1–2.2)
		Unsafe	2.2 (1.8–2.6)	1.5 (0.8–2.6)	2.4 (2.0-2.8)							1.8 (1.5–2.1)	1.1 (0.6–2.3)	2.1 (1.8–2.4)

PR = Prevalence ratios

95%CI = 95% confidence intervals

Models 1: Different models with the bivariate association between each explanatory variable and poor self-rated health

Model 2: Multivariate association between economic resources variables and poor self-rated health

Model 3: Multivariate association between economic resources variables. social support and poor self-rated health

Model 4: Multivariate association between all explanatory variables and poor self-rated health

**Table 5:** Association between self-perceived poor health and social. physical and safety environment variables. women according to educational level. Chile 2016

				Model 1			Model 2			Model 3			Model 4	
			**Women**	**Women with low educational level**	**Women with medium/high educational level**	**Women**	**Women with low educational level**	**Women with medium/high educational level**	**Women**	**Women with low educational level**	**Women with medium/high educational level**	**Women**	**Women with low educational level**	**Women with medium/high educational level**
			PR (95%CI)	PR (95%CI)	PR (95%CI)	PR (95%CI)	PR (95%CI)	PR (95%CI)	PR (95%CI)	PR (95%CI)	PR (95%CI)	PR (95%CI)	PR (95%CI)	PR (95%CI)
*Social Environment*	Sense of Belonging to the neighborhood	Agree	1	1	1	1	1	1				1	1	1
		Neither Agree nor Disagree	1.2 (1.0-1.4)	1.1 (0.9–1.3)	1.2 (1.0-1.5)	1.1 (0.9–1.4)	1.1 (0.9–1.4)	1.1 (0.8–1.5)				1.1 (0.9–1.3)	1.1 (0.9–1.3)	1.0 (0.8–1.3)
		Disagree	1.5 (1.2–1.9)	1.6 (1.3–1.9)	1.3 (1.2–1.5)	1.4 (1.0-1.8)	1.6 (1.2-2.0)	1.1 (0.9–1.4)				1.2 (0.9–1.5)	1.5 (1.2–1.8)	1.0 (0.8–1.1)
	Perception of Support Networks	Agree	1	1	1	1	1	1				1	1	1
		Neither Agree nor Disagree	1.2 (1.0-1.4)	0.8 (0.6–1.1)	1.3 (1.2–1.6)	1.1 (0.8–1.3)	0.8 (0.5–1.1)	1.2 (1.0-1.5)				1.1 (0.9–1.4)	0.8 (0.5–1.1)	1.2 (1.0-1.5)
		Disagree	1.5 (1.3–1.8)	1.2 (1.0-1.5)	1.6 (1.3–1.9)	1.4 (1.1–1.7)	1.1 (0.9–1.3)	1.5 (1.2–1.9)				1.3 (1.0-1.6)	1.0 (0.8–1.3)	1.4 (1.1–1.7)
	Egotropic Participation	Yes participates	1	1	1	1	1	1				1	1	1
		Does not participate	1.3 (1.1–1.5)	1.0 (0.8–1.2)	1.5 (1.2-2.0)	1.2 (1.1–1.4)	1.0 (0.9–1.1)	1.4 (1.2–1.7)				1.2 (1.1–1.3)	1.0 (0.9–1.2)	1.3 (1.1–1.6)
	Sociotropic Participation	Yes participates	1	1	1	1	1	1				1	1	1
		Does not participate	1.0 (0.9–1.1)	1.0 (0.8–1.2)	1.1 (0.9–1.2)	0.9 (0.8–1.1)	1.0 (0.8–1.2)	1.0 (0.8–1.1)				1.0 (0.8–1.1)	1.0 (0.8–1.2)	1.0 (0.8–1.2)
	Religious Participation	Yes participates	1	1	1	1	1	1				1	1	1
		Does not participate	1.2 (0.8–1.9)	1.1 (1.0-1.3)	1.6 (0.7–3.5)	1.2 (0.8–1.9)	1.1 (0.9–1.3)	1.4 (0.6–3.2)				1.2 (0.8–1.8)	1.1 (0.9–1.3)	1.4 (0.7-3.0)
*Physical Environment*	Perception of problems with Quality of public space	No problem	1	1	1				1	1	1	1	1	1
		Yes Problem	1.4 (1.3–1.6)	1.1 (1.1–1.2)	1.6 (1.4–1.8)				1.3 (1.2–1.3)	1.1 (1.0-1.2)	1.4 (1.2–1.5)	1.2 (1.1–1.3)	1.0 (0.9–1.1)	1.3 (1.2–1.5)
	Perception of pollution problems in the environment	No problem	1	1	1				1	1	1	1	1	1
		Yes Problem	1.6 (1.3–1.9)	1.3 (1.1–1.6)	1.6 (1.1–2.2)				1.4 (1.1–1.7)	1.3 (1.1–1.6)	1.4 (1.0–2.0)	1.3 (1.0-1.6)	1.2 (1.0-1.4)	1.3 (0.9–1.8)
	Perception of problem of infrastructure availability in the neighborhood	No problem	1	1	1				1	1	1	1	1	1
		Yes Problem	1.3 (1.2–1.4)	1.1 (1.0-1.3)	1.3 (1.1–1.5)				1.1 (0.9–1.2)	1.0 (0.9–1.2)	1.1 (0.9–1.3)	1.1 (0.9–1.2)	1.0 (0.9–1.2)	1.0 (0.9–1.3)
*Safety*	Perceived safety in the neighborhood	Safe	1	1	1							1	1	1
		Somewhat safe	1.3 (1.0-1.5)	1.3 (1.1–1.6)	1.3 (1.1–1.6)							1.1 (0.9–1.4)	1.3 (1.0-1.7)	1.1 (0.9–1.3)
		Unsafe	1.6 (1.4–1.8)	1.6 (1.3–1.9)	1.6 (1.4–1.9)							1.3 (1.0-1.5)	1.4 (1.1–1.7)	1.3 (1.0-1.6)

PR = Prevalence ratios

95%CI = 95% confidence intervals

Models 1: Different models with the bivariate association between each explanatory variable and poor self-rated health

Model 2: Multivariate association between economic resources variables and poor self-rated health

Model 3: Multivariate association between economic resources variables. social support and poor self-rated health

Model 4: Multivariate association between all explanatory variables and poor self-rated health

In women (Table 5) in model 1, the factors associated with the higher probability of poor SPH in the social environment were the *feeling of not belonging* (PR = 1.5; CI = 1.2–1.9), *not perceiving support networks* (PR = 1.5; CI = 1.3–1.8), *egotropic non-participation* (PR = 1.3; CI = 1.1-1. 5), *perceived problems with the quality of space* (PR = 1.4; CI = 1.3–1.6), *perceived problems with pollution* (PR = 1.6; CI = 1.3–1.9), *perceived problems with infrastructure availability* (PR = 1.3; CI = 1.2–1.4), and *perceived unsafety* (PR = 1.6; CI = 1.4–1.8). These associations were maintained when we stratified by educational level, although with greater statistical significance in women with medium/higher education. The associations were maintained in Model 2 (all social environment variables). When we analyzed all the physical environment variables (model 3), the association between poor SPH and the *perception of infrastructure availability problems* was lost. When we introduced all the variables (model 4) in general the associations with poor SPH in women were maintained. When we stratified by educational level, medium/highly educated women who reported *no perceived support networks* (PR = 1.4, CI = 1.1–1.7), *no egotopic involvement* (PR = 1.3, CI = 1.1–1.6) and *no perceived problems with the quality of space* (PR = 1.3, CI = 1.2–1.5) were at higher probability of poor SPH. While in women with a lower educational level, poor SPH was associated with a *feeling of not belonging* (PR = 1.5; CI = 1.2–1.8) and *perceived problems with pollution* (PR = 1.2; CI = 1.0-1.4). There was association between poor SPH and perceived unsafety (PR = 1.3, CI = 1.0-1.5) at both levels of education.

### Sensitivity analysis

To validate our results, we repeated all the analyses with the variable “Satisfaction with life”, which is an indicator of quality of life. [29] The results were similar to our main findings, with greater associations among women and people with a higher level of education (data not shown).

## Discussion

Our results show the association between the physical, social and safety elements of the neighborhood environment and SPH in urban settings in Chile. First, women had worse SPH than men, especially those with lower educational attainment. In men with a higher educational level, the poor health was associated with the feeling of not belonging to the neighborhood and the feeling of unsafety. In contrast, in men with a lower educational level, no statistically significant association was found, although the association between poor SPH and lack of support networks and feeling of not belonging was almost statistically significant. In women with a higher education level, poor health was associated with perceived lack of support networks, not participating in egotropic social organizations, and identifying problems in the quality of the environment. In women with a lower educational level, poor SPH was associated with a lack of a sense of belonging to the neighborhood and perceiving pollution problems. Unsafety was a risk factor for poor SPH in women, irrespective of educational level.

A gender perspective analysis of the data suggests that women’s health is associated with most neighborhood elements. The reasons could be due to the different ways in which men and women experience public spaces, associated with the gender-based division of labor. First, current urban planning prioritizes the construction and organization of public space for productive work and leisure, predominantly performed by men, to the detriment of care work, predominantly performed by women [30]. Although care work is carried out in private spaces, neighborhood spaces are also used to perform daily tasks. Therefore, women spend more time in the public spaces of the neighborhood. This can be made more difficult if the spaces are created for the development of productive work, for example, by not having sidewalks in good condition to move around with the shopping cart or baby carriage, which can generate more stress, for example, among women. This would explain why, in relation to the physical environment, the associations of poor health would be more closely related with the quality of the space, such as problems with sidewalks or lighting, and with pollution, such as micro-garbage dumps, which have been described in the literature [12,30], as they make daily life more difficult. Because women spend more time in the neighborhood, they may see the community as an extension of the home [31]. This could explain why our results show stronger associations between poor health and elements of the social environment such as lack of support networks, not belonging to the neighborhood or lack of social participation, which is similar to findings reported in the literature [12].

The poor health in men was mainly due to elements of the physical environment, such as the perceived lack of availability of infrastructure in the neighborhood, i.e., sports facilities [32,33]. From a gender perspective, this may be explained by the fact that men mostly use public spaces for leisure with their peers. In this regard, the perception of problems in this environment may be associated with worse health. In addition, the poor health in men was associated with the feeling of not belonging to the neighborhood. This association could be due to other reasons. Studies have shown the positive health effects of a sense of belonging in men, who feel connected to the neighborhood environment by living in the same place as previous generations [34], compared to women. In this sense, the sense of belonging would have to do with personal identity and family history [35], rather than an indicator of strengthening social networks, as in the case of women.

In relation to differences by socioeconomic position, both men and women with low educational level had the worst perceived health and the highest perceptions of problems in all the environments analyzed. Furthermore, the associations between poor health and elements of the neighborhood were less significant, especially in men with fewer years of schooling. Explanations in the literature on the effect of neighborhood environments on people’s health have mostly focused on ecological analysis of socioeconomic position, and how living in an area with greater deprivation has worse health effects [36,37]. Studies analyzing the effects of socioeconomic position at the individual level are rather scarce. Even so, our results follow the trend of these investigations. It has been found that, at the individual level, people with a lower socioeconomic position take less advantage of recreational facilities, even if available in their neighborhoods [38], and benefit less from green areas [39], compared with people with a higher socioeconomic position. This could suggest that cultural, social or normative factors have an influence on the use of the facilities. In this way, for example, the benefits of these stress-reducing factors could be limited. However, the SPH of people with lower socioeconomic position, tended to be associated with elements of the social environment. This means that not having a support network or a sense of belonging has a greater impact on their health, as opposed to the conditions or material resources available in the neighborhood. One hypothesis could be that in the face of material precariousness (individual or neighborhood), the social can become relevant in daily survival [40]. Finally, studies analyzing health and socioeconomic position from multilevel models in Chile [41], have highlighted the need for analysis that includes the contextual socioeconomic level, which currently do not exist in the country.

A feeling of unsafety was also one of the variables most closely associated with poor SPH. In Chile, although citizen security is considered one of the best in Latin America, and similar to that prevailing in European countries, opinion polls indicate that the population has a growing sense of unsafety linked to criminal violence and to the economic and political-institutional system [42]. In relation to health, scientific studies show complex relationships associated with mental health problems [11]. Fear related to insecurity can lead to avoidant behaviors (e.g., limiting movement outside the home), which can have a negative impact on physical activity and social interaction [11]. Our results showed a strong association between unsafety and poor SPH, but the cause of the unsafety and associated fear differed by gender and socioeconomic position. In men, the cause was related to possessions or assets, while in women, it was related to their bodies and the sexual sphere [43]. In addition, in Chile, it has been reported that for people with lower socioeconomic status, representations of unsafety are based on the perception of an unequal and unfair environment [44,45]. In contrast, for the inhabitants of more affluent neighborhoods, unsafety is a process perceived as external [44], predominantly associated with the fear of being victims of assaults or robberies [45].

Our study has some limitations. First, the low number of people in some subgroups, for example, men and women with low educational level, may have influenced the detection of statistically significant associations due to the low statistical power of the data; nevertheless, some statistically significant associations were detected in these population groups. Second, although the study was conducted from a gender perspective, the survey only included the binary variable “sex”, which, although not interchangeable [46,47], was used as a proxy for gender. This implies two limitations: first, the assumption that gender identity develops in correspondence with sexual anatomy; and second, the assumption that gender is binary. Both assumptions are based on the patriarchal system, and surveys only very rarely allow us to analyze the results beyond these assumptions. And third, the results are representative of the urban areas of the country, which do not take into consideration the particularities of each city, but this first approximation is relevant to understand factors related to urban and social elements with health.

As a strength, to our knowledge, this study is the first, to evaluate elements of the urban neighborhood environment with SPH in Chile. This is relevant because these factors are currently susceptible to intervention to improve the quality of life of the population, for example, through urban regeneration programs. Therefore, the evidence from this study could help to improve the planning of these policies, taking health into account. In addition, this is one of the few studies that analyzes the intersection between gender and socioeconomic position, which should be understood bearing in mind the complexity of both axes. Future studies should focus, ideally from a more qualitative perspective, on understanding gender differences and their different socioeconomic positions, to deepen the concepts and understand the mechanisms by which neighborhood elements are related to health.

## Conclusions

Self-perceived health is associated with elements of the neighborhood, showing differences according to gender and socioeconomic position. Poor health in women was associated with perceived problems or deficiencies in most elements of the neighborhood. In men, poor health was mostly associated with elements perceived to be in poor condition or with problems in the physical environment. Poor health among those with less education had little association with neighborhood elements. Insecurity was associated with poor health in both women and men. Interventions around these elements in the neighborhoods become tools that can improve the health of the population, which should be carried out and evaluated considering the axes of inequality.

## Data Availability

The database belongs to the Department of Epidemiology of the Ministry of Health of Chile and the survey is open to the general public (http://epi.minsal.cl/bases-de-datos/).
